# Optogenetic control of epithelial-mesenchymal transition in cancer cells

**DOI:** 10.1038/s41598-018-32539-3

**Published:** 2018-09-20

**Authors:** Xiaoxu Zhou, Jian Wang, Junye Chen, Yuankai Qi, Di Nan, Luhong Jin, Xiaohan Qian, Xinyi Wang, Qingyong Chen, Xu Liu, Yingke Xu

**Affiliations:** 1 Department of Biomedical Engineering, Key Laboratory of Biomedical Engineering of Ministry of Education, Zhejiang Provincial Key Laboratory of Cardio-Cerebral Vascular Detection Technology and Medicinal Effectiveness Appraisal, Zhejiang University, Hangzhou, 310027 China; 2 Department of Respiratory Oncology, The 117th Hospital of PLA, Hangzhou, 310013 China; 3 Department of Optical Engineering, State Key Laboratory of Modern Optical Instrumentation, Zhejiang University, Hangzhou, 310027 China; 4 Department of Hepatobiliary and Pancreatic Surgery, the Second Affiliated Hospital, School of Medicine, Zhejiang University, Hangzhou, 310009 China; 5 Department of Endocrinology, The Affiliated Sir Run Run Shaw Hospital, Zhejiang University School of Medicine, Hangzhou, 310016 China

## Abstract

Epithelial-mesenchymal transition (EMT) is one of the most important mechanisms in the initiation and promotion of cancer cell metastasis. The phosphoinositide 3-kinase (PI3K) signaling pathway has been demonstrated to be involved in TGF-β induced EMT, but the complicated TGF-β signaling network makes it challenging to dissect the important role of PI3K on regulation of EMT process. Here, we applied optogenetic controlled PI3K module (named ‘Opto-PI3K’), which based on CRY2 and the N-terminal of CIB1 (CIBN), to rapidly and reversibly control the endogenous PI3K activity in cancer cells with light. By precisely modulating the kinetics of PI3K activation, we found that E-cadherin is an important downstream target of PI3K signaling. Compared with TGF-β treatment, Opto-PI3K had more potent effect in down-regulation of E-cadherin expression, which was demonstrated to be regulated in a light dose-dependent manner. Surprisingly, sustained PI3K activation induced partial EMT state in A549 cells that is highly reversible. Furthermore, we demonstrated that Opto-PI3K only partially mimicked TGF-β effects on promotion of cell migration *in vitro*. These results reveal the importance of PI3K signaling in TGF-β induced EMT, suggesting other TGF-β regulated signaling pathways are necessary for the full and irreversible promotion of EMT in cancer cells. In addition, our study implicates the great promise of optogenetics in cancer research for mapping input-output relationships in oncogenic pathways.

## Introduction

Epithelial-mesenchymal transition (EMT) is a physiological process that plays an important role in embryonic development, wound healing and stem cell behavior^1^. It is also induced in certain pathological conditions, such as fibrosis and cancer metastasis. The process of EMT is advocated to be involved in tumor progression and the acquisition of metastatic potential of human cancers^2^. Therefore, the study of EMT induction and its underlying regulatory signaling pathways are of great therapeutic interest in the treatment of cancer.

Transforming growth factor-β (TGF-β) induces EMT in many types of cancer cells, which is achieved through the regulation and cooperation between many different signaling pathways^2,3^. It has been demonstrated that TGF-β stimulation activates both Smad and non-Smad signaling pathways (e.g. PI3K/Akt, MAPK) that are actively involved in regulation of EMT process. Furthermore, other signaling pathways, such as Wnt, Notch and Hedgehog signaling have also been shown to play a role in regulation of EMT in cultured cancer cells^3,4^. Thus, the complex signaling networks and crosstalk between different signaling pathways make it challenging to dissect the important role of individual signaling molecules in this regulated process of EMT induction.

Recent advances in optogenetics offer tunable methods to modulate the kinetics of signaling transduction that permit for mapping cellular input-output relationships^5–7^. Compared with chemical regulated systems, light does not interfere with cellular signaling pathways and is easily delivered, which enables rapid, reversible and quantitative manipulation of intracellular activities^6^. Here, we applied optogenetic controlled PI3K module, namely ‘Opto-PI3K’ to precisely control the activation of endogenous PI3K in cultured cancer cells. By precisely modulate the kinetics of PI3K activation, we found that E-cadherin is an important downstream target of PI3K signaling, and Opto-PI3K down-regulates E-cadherin expression in a light dose-dependent manner. We further demonstrated that light-activated PI3K induced partial and reversible EMT in A549 cells. Our established method of quantitative optogenetic control of intracellular PI3K signaling may have vast applications in studying of other unidentified new roles of PI3K in cancer biology.

## Materials and Methods

### Materials and DNA constructs

The rabbit anti-total Akt, anti-phospho-Akt(308), anti-phospho-Akt(473), anti-phospho-Smad2, anti-E-cadherin, anti-vimentin, anti-Snail1 and mouse anti-GAPDH antibodies were purchased from Cell Signaling Technology (Danvers, MA). The mouse anti-Zeb1 antibody was ordered from Santa Cruz Biotechnology (Santa Cruz, CA). Antibodies against rabbit IgG and mouse IgG conjugated with horseradish peroxidase were purchased from Abcam (Cambridge, MA). Fluorescent-labeled secondary antibodies were from Molecular Probes (Life Technologies, Carlsbad, CA). Common chemicals and reagents were purchased from Sigma-Aldrich unless otherwise noted. Plasmids encoding CIBN-GFP-CAAX, CIBN-CAAX, mCherry-CRY2-iSH2, CRY2-iSH2 and PH-Akt-mRFP were generated as described previously^8,9^.

### Cell culture, transfection and LED illumination

A549 human lung cancer cells were cultured in Ham’s F-12 media (Life Technologies) with 10% fetal bovine serum (FBS, Gibco, Gland Island, NY) and 1% penicillin G/streptomycin. HeLa cells were cultured in Dulbecco’s modified Eagle’s media (DMEM, Life Technologies) with 10% fetal bovine serum (FBS) and 1% penicillin G/streptomycin. The cells were kept at 37 °C in a humidified 5% CO_2_ incubator. Cells were transfected with indicated plasmids using the Lipofectamine 2000 (Life Technologies) according to the manufacturer’s instructions. For optogenetic experiments, the cells transfected with plasmids encoding light-sensitive modules were illuminated with a custom-designed light-emitting diode (LED) array whose light intensity (maximum illumination doses of 20 mW/cm^2^; 1% of maximum light intensity at 0.2 mW/cm^2^ was used for prolonged illumination) and ON/OFF frequency can be controlled remotely with programmed software^8^.

### Migration assay

Transwell migration assay and scratch-wound assay were performed as described previously^10^. HeLa cells were transfected with optogenetic constructs and cultured in DMEM with 1% FBS in the upper chamber of 8-μm pore transwell (Corning Costar, Cambridge, MA). The cells were incubated with or without 10 ng/ml of TGF-β (PeproTech, Rocky Hill, NJ) in DMEM with 10% FBS and planted in a 24-well lower chamber. After 6 h of transfection, the cells were illuminated with blue-light LED array (0.2 mW/cm^2^), non-migrated cells were removed with cotton swab and the cells that had migrated through pores were stained with Hoechst dye (Life Technologies, Carlsbad, CA). Images of eight fields per insert were taken with Olympus IX81 fluorescence microscopy (Olympus, Tokyo, Japan).

For scratch-wound assay, confluent A549 cells were scratched with 10 μl pipette tip and the cells were cultured in Ham’s F-12 media (Life Technologies) with 5% FBS for migration assay. As a positive control, 10 ng/ml of TGF-β was added to the scratched cells to promote cell migration. A series of images were captured at the beginning and at different time intervals during cell migration to close the wound. For optogenetic experiments, the transfected cells were illuminated with blue-light LED array (0.2 mW/cm^2^) in the incubator. For quantification, the wound area was examined by the wound margin that was calculated using the NIH ImageJ 1.45 s software (NIH, Bethesda, MD, USA) and normalized with the start points.

### Immunostaining and biochemical methods

Immunofluorescence cell staining was performed as described previously^8^. A549 cells were fixed with 3.7% paraformaldehyde in phosphate-buffered saline (PBS) for 10 min at room temperature. Cells were permeabilized with 0.1% Triton X-100 for 5 min, and blocked with 1% bovine serum albumin (BSA) and 3% normal goat serum in PBS for 45 min. The Akt phosphorylation was detected using rabbit anti-phospho-Akt antibodies (1:200 dilution; Cell Signaling Technology) followed by Cy3-conjugated anti-rabbit IgG (1:200 dilution). The E-cadherin was detected using rabbit anti-E-cadherin antibody (1:200 dilution; Cell Signaling Technology) followed by Alexa Fluor 546-conjugated anti-rabbit secondary antibody (1:200 dilution). Finally, the cells were washed, mounted and imaged with Epifluorescence microscopy or total internal reflection fluorescence microscopy (TIRFM) (see below).

Biochemistry experiments were performed as previously described^8,11^. Briefly, cells were washed with PBS and lysed in RIPA lysis buffer (25 mM Tris, pH 7.4, 150 mM NaCl, 1 mM EDTA, 1% Triton X-100) containing protease inhibitors (Complete; Roche). Equal amounts of protein (quantified by BCA protein assays, Thermo Scientific) in each sample were analyzed by SDS-PAGE and immunoblotting. Proteins were transferred to polyvinylidene difluoride membrane (Bio-Rad) and hybridized to an appropriate primary antibody and horseradish peroxidase-conjugated secondary antibody for subsequent detection with ECL (Millipore). Densitometric analysis was performed with ImageJ software (NIH, USA).

### TIRF Microscopy

A custom-built multi-angle TIRFM system was used to image the cells^12,13^. This TIRFM setup was based on an Olympus IX-70 microscope, equipped with 405-, 488- and 568-nm laser lines, a 60 × 1.49 NA TIRF objective (Olympus) and an EMCCD camera (iXon887; Andor Technology). The angles of the illumination laser beam can be rapidly adjusted by two-dimensional galvometers under the control of custom-written software, which allows for both Epi- and TIRF imaging of cells. For imaging of live cells, TIRF images were acquired under the control of Andor iQ software at 0.2–0.5 Hz with exposure times in the rage of 200–500 ms.

### Image analysis and statistics

Stacks of time-lapse images were processed and analyzed using ImageJ 1.45 s (NIH, USA). For quantitative analysis of the dynamics of fluorescence increase at the cell surface after optogenetic activation, the intensities of the footprint of the cells under the TIRF field were subtracted for background and normalized with intensities before the activation. For immunofluorescence staining images, the average intensity of the fluorophores was normalized by background subtraction. Images were prepared with Photoshop (Adobe). Unless otherwise indicated, all data are presented as the mean ± SEM, and were analyzed using a Student’s *t*-test. *P* values less than 0.05 were considered significant, and * indicates *P* < 0.05; ** indicates *P* < 0.01; *** indicates *P* < 0.001.

## Results and Discussion

### PI3K signaling is necessary for TGF-β induced EMT in A549 cells

**Figure 1:**
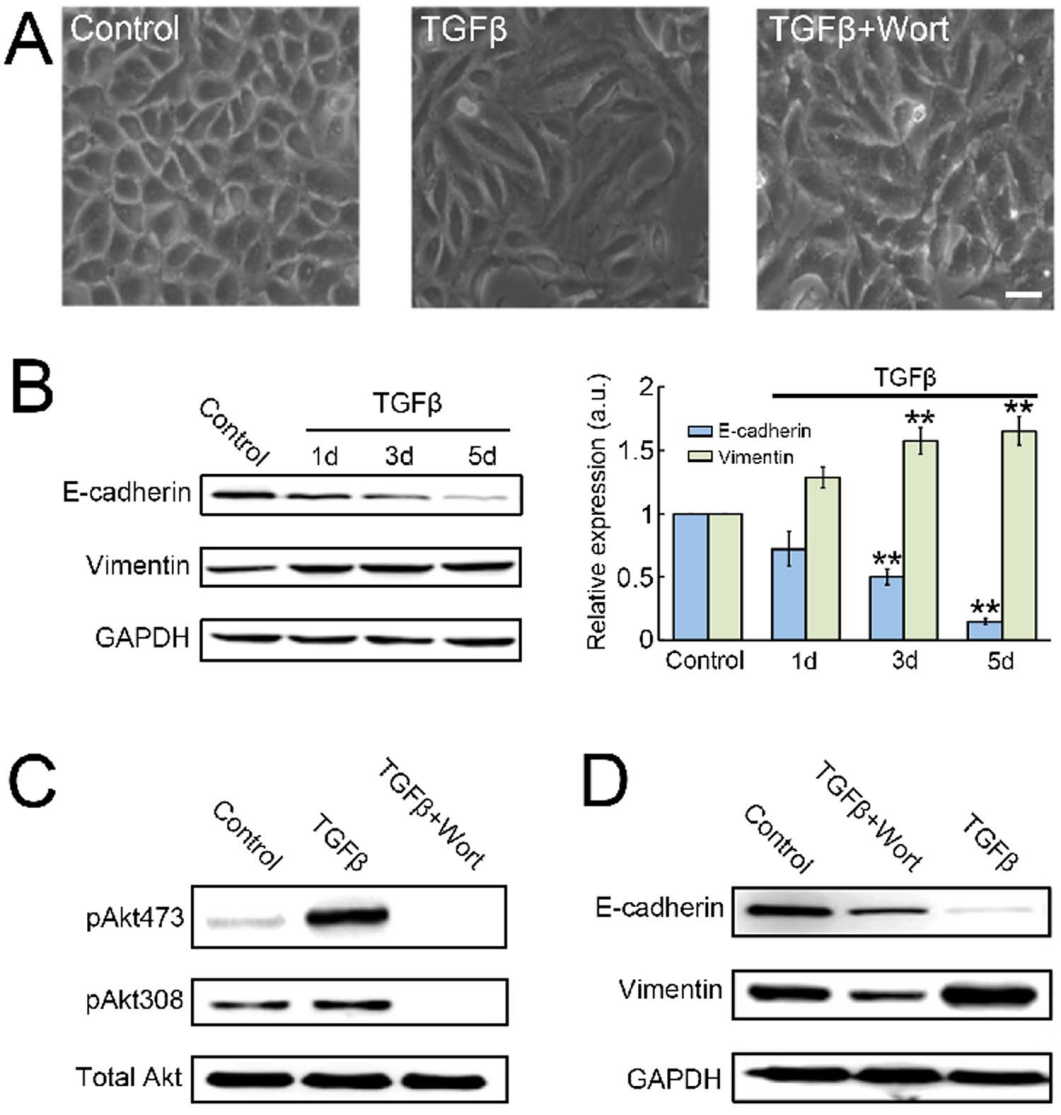
PI3K activity is required for TGF-β induced EMT in A549 cancer cells. (**A**) A549 cancer cells were incubated with or without TGF-β (10 ng/ml), or in combination of wortmannin (100 nM) treatment for 24 h. DIC images show changes in cell morphology in response to TGF-β. Scale bar, 10 μm. (**B**) Immunoblot analysis of E-cadherin and vimentin expressions in control A549 cells and cells treated with 10 ng/ml TGF-β as time indicated (left); the ratios of E-cadherin and vimentin to GAPDH abundance are indicated for each sample, normalized to control cells (right). Data from four independent experiments; **P < 0.01 vs control. (**C**) Immunoblot analysis of Akt phosphorylation in control A549 cells and cells treated with 10 ng/ml TGF-β alone or with 100 nM wortmannin for 24 h. (**D**) Immunoblot analysis of E-cadherin and vimentin expressions in control A549 cells and cells treated with 10 ng/ml TGF-β alone or with 100 nM wortmannin for 24 h. Displayed blots are representative results of at least three independent experiments.

We first investigated the importance of PI3K signaling on TGF-β induced EMT in human alveolar basal epithelial cell line, A549. The A549 cells were treated with 10 ng/ml of TGF-β, which has been shown to induce fully EMT process^14,15^. After 24 h of TGF-β treatment, A549 cells started to display dramatic morphological changes from cuboidal to an elongated spindle shape (Fig. 1A), whereas the two EMT characteristic markers E-cadherin (E-cad) and vimentin expressions were not significant different compared with untreated cells (Fig. 1B). Similar to previously reported studies^14^, we found that TGF-β treatment induced the down-regulation of epithelial marker E-cad expression and up-regulated expression of mesenchymal marker vimentin in a time-dependent manner (Fig. 1B). Furthermore, significant changes in these protein expressions compared with untreated cells were only detected after prolonged incubation of TGF-β for several days (Fig. 1B). Surprisingly, all these TGF-β induced cellular effects were significantly inhibited by co-treatment of cells with 100 nM of wortmannin (Fig. 1A–D), suggesting that an active PI3K signaling (Fig. 1C) is necessary for TGF-β induced EMT on A549 cells. Taken together, our results are consistent with previous studies, which demonstrated that PI3K activity is required for TGF-β mediated EMT in cultured cancer cells^16–18^.

### Using light to quantitatively control the PI3K activation in A549 cells

**Figure 2:**
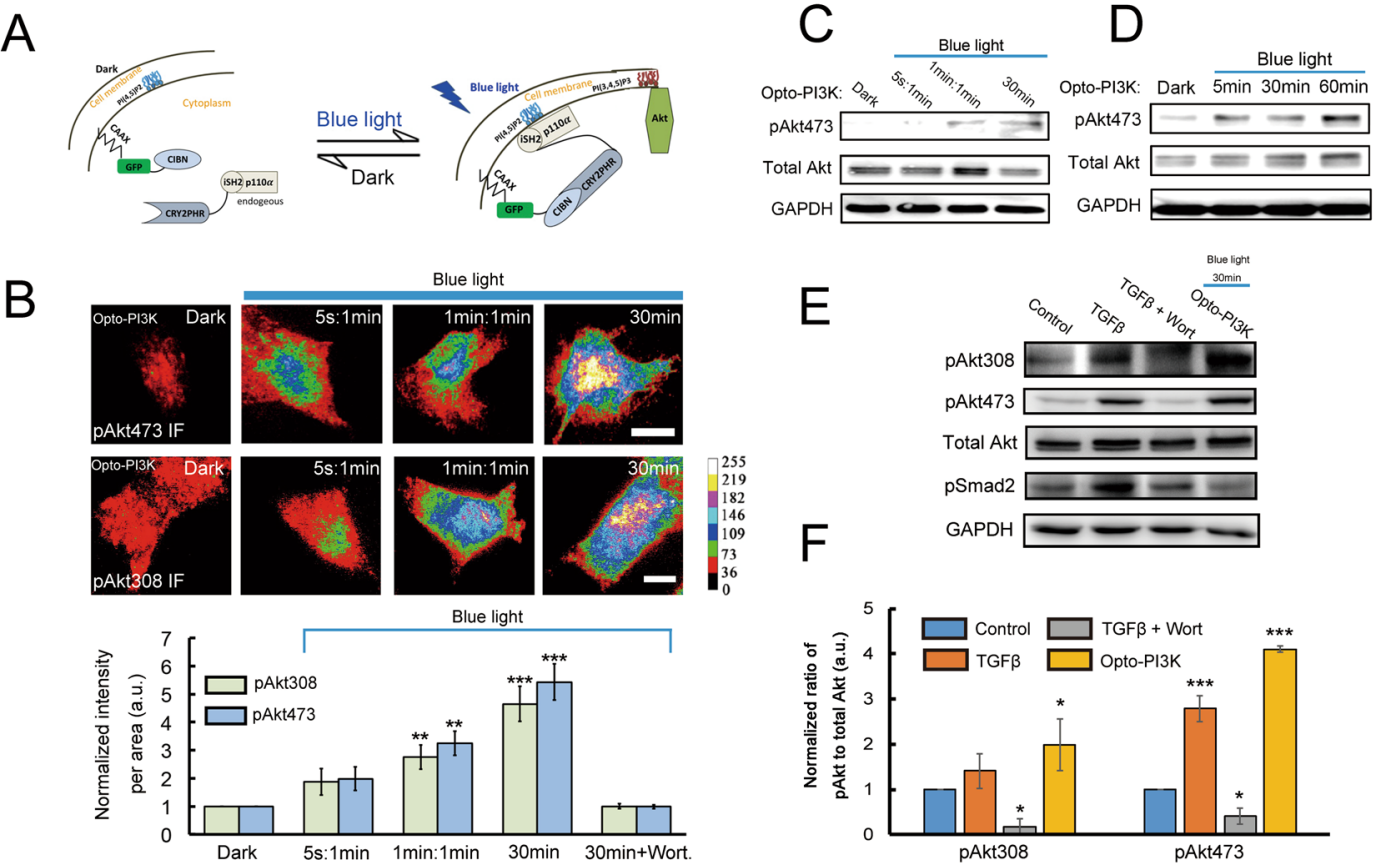
Tunable activation of PI3K signaling in A549 cancer cells by light. (**A**) Schematic drawing depicting constructs used to activate PI3K using optogenetics. (**B**) Optogenetic control of endogenous Akt phosphorylation in a light dose-dependent manner. A549 cells were transfected with Opto-PI3K constructs. After 18–24 h of transfection, the cells were illuminated with blue-light LED array (0.2 mW/cm^2^) for total of 30 min with different ON/OFF frequencies (5 s: 1 min means light ON for 5 s, and then OFF for 1 min; 1 min: 1 min means light ON for 1 min, and then OFF for 1 min; 30 min means light ON for 30 min). After 30 min of activation, the cells were fixed and labeled for Akt phosphorylation at both Ser473 and Thr308 residues. Immunofluorescence staining of pAkt was imaged by TIRFM and quantified. (*n* = 50 cells, data are mean ± SEM). **P < 0.01 vs control; ***P < 0.001 vs control; Scale bar, 10 μm. (**C**,**D**) Immunoblot analysis of pAkt473 in A549 cells transfected with Opto-PI3K constructs or with and without exposure to blue-light LED array (0.2 mW/cm^2^) illumination with different ON/OFF frequencies (**C**) or with different exposure times. (**D**) Displayed blots are representative results of at least three independent experiments. (**E**,**F**) Immunoblot analysis of Akt phosphorylation and Smad2 phosphorylation (pSmad2) in A549 cells transfected with Opto-PI3K constructs or treated with 10 ng/ml TGF-β alone or with 100 nM wortmannin for 30 min (**E**) and quantified. (**F**) Displayed blots are representative results of at least three independent experiments. Data are mean ± SEM. *P < 0.05 vs control; ***P < 0.001 vs control.

To be able to rapidly and precisely regulate the PI3K activity, we firstly designed and tested the function of ‘Opto-PI3K’, the light activated PI3K optogenetic module on A549 cells. Our optogenetic system is based on *Arabidopsis thaliana* cryptochrome 2 (CRY2) and the transcription factor CIBN, whose heterodimerization can be reversibly modulated with blue-light illumination^8,19^. The ‘Opto-PI3K’ module constitutes the CAAX-tagged CIBN that localizes on the plasma membrane (PM), and the cytosolic expressed CRY2-iSH2 that binds constitutively to the endogenous PI3K (Fig. 2A). Upon blue-light illumination, the cytosolic CRY2-iSH2 proteins mobilize PI3K to the cell surface, which promotes the conversion of PI(4,5)P_2_ to PI(3,4,5)P_3_ on the PM and recruits and activates Akt (Fig. 2A). We have previously demonstrated that this optogenetic module is able to activate PI3K signaling and to induce downstream Akt phosphorylation in adipocytes at the presence of blue-light illumination^8^. Here, we sought to study whether it is feasible to quantitatively control the PI3K activity by tunable light activation.

Firstly, we demonstrated that by illuminating single A549 cells with different doses of 488 nm laser using the total internal reflection fluorescence microscopy (TIRFM), the protein heterodimerization between CIBN and CRY2, and the induced production of PI(3,4,5)P_3_ by Opto-PI3K as evaluated by expressed PH-Akt-mRFP on the cell surface were regulated in a light dose-dependent manner (Supplementary Figs S1 and S2). These indicate the feasibility of tunable control intracellular PI3K signaling by light. Secondly, we used custom-designed blue-light LED array to activate Opto-PI3K expressing A549 cells, and studied the downstream Akt phosphorylation under different light conditions. We applied three different light doses from low, medium to high by using different ON/OFF light cycles in total of 30 min of illumination (5 s: 1 min means light ON for 5 s, and then OFF for 1 min; 1 min: 1 min means light ON for 1 min, and then OFF for 1 min; 30 min means light ON for 30 min). The A549 cells were fixed and stained for Akt phosphorylation (pAkt) after light treatment. The TIRFM images and quantification results shown on Fig. 2B illustrated that Akt phosphorylation on both Ser473 and Thr308 residues at the PM were evaluated in a light dose-dependent manner (Fig. 2B). In addition, our biochemical results further validated the correlative relationships between the time and doses of illuminating blue-light and the amount of activated Akt in A549 cells (Fig. 2C,D). Furthermore, we showed that illumination of non-transfected control cells with blue-light LED array (0.2 mW/cm^2^) for 30 min had no effect on Akt phosphorylation, suggesting that the increases of pAkt in A549 cells were induced by Opto-PI3K (Fig. S3A). It has been recently shown that sustained blue-light illumination induced cell damage in primary cultured neuronal cells^20^, but in A549 cells we did not detect apparent effects on cell function (Fig. S3).

In addition, we performed experiments to compare the differences between TGF-β and Opto-PI3K activated Akt phosphorylation in A549 cells. The control cells were treated with or without 10 ng/ml TGF-β, or together with 100 nM wortmannin for 30 min. Opto-PI3K transfected A549 cells were illuminated with blue-light LED array (0.2 mW/cm^2^) for 30 min. As shown on the immunoblotting results in Fig. 2E and quantified in Fig. 2F, Opto-PI3K potently activated downstream Akt phosphorylation to the levels similar as TGF-β treatment (Fig. 2E,F). Of note, the phosphorylation of Smad2 protein was only detected in TGF-β stimulated A549 cells, but not in Opto-PI3K expressing cells, suggesting the specificity of Opto-PI3K on activation of non-Smad signaling pathway.

Taken together, these results indicated that we were able to employ Opto-PI3K light-sensitive module to acutely, specifically and quantitatively control the PI3K activation in A549 cancer cells. Although similar approaches have been previously reported to finely control the kinetics of Erk signaling transduction or to regulate gene expression in cells^7,21^, our study demonstrated in both single cell and bulky biochemical assays that Opto-PI3K permits tunable control of PI3K signaling in cultured cancer cells.

### Sustained PI3K activation by light induces EMT and promotes cell migration in cultured cancer cells

**Figure 3:**
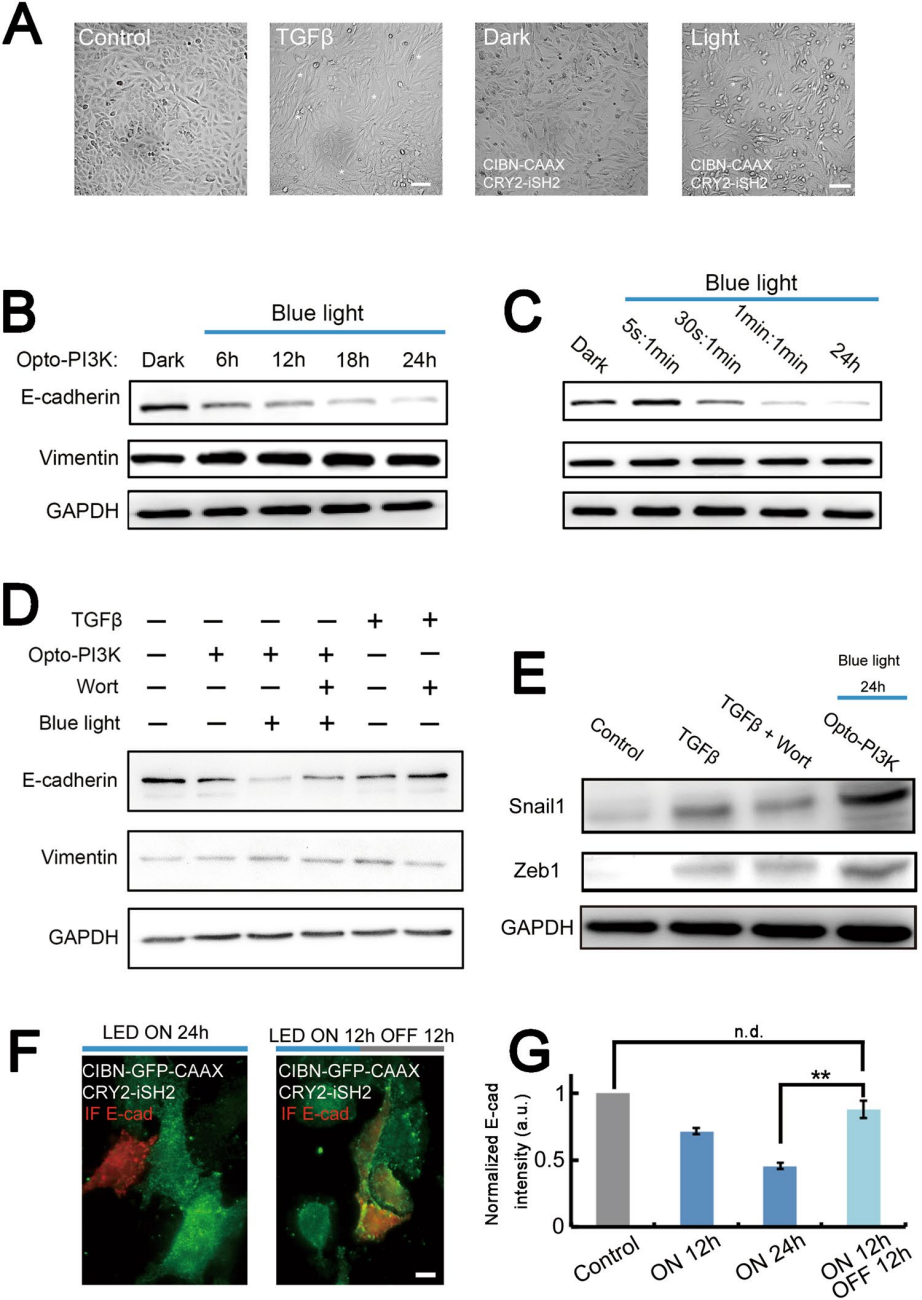
Sustained PI3K activation by light induces EMT and promotes cell migration in cultured cancer cells. (**A**) A549 cancer cells were transfected with Opto-PI3K constructs. The cells were then activated with or without exposure to blue-light LED array (0.2 mW/cm^2^) for 24 h. DIC images show changes in cell morphology in response to light activation. Scale bar, 100 μm. (**B**,**C**) Immunoblot analysis of E-cadherin and vimentin expressions in A549 cells transfected with Opto-PI3K constructs, and activated with or without exposure to different doses of blue-light LED array illumination (0.2 mW/cm^2^). Different exposure times to blue-light (**B**) and different ON/OFF frequencies of blue-light exposure in total of 24 h (5 s: 1 min means light ON for 5 s, and then OFF for 1 min; 30 s: 1 min means light ON for 30 s, and then OFF for 1 min; 1 min: 1 min means light ON for 1 min, and then OFF for 1 min; 24 h means light ON for 24 h) (**C**). (**D**) Compare the effects of Opto-PI3K and TGF-β (10 ng/ml) in regulation of EMT markers expression. The E-cadherin and vimentin expressions were analyzed by immunoblot on the conditions indicated. (**E**) Immunoblot analysis of Snail1 and Zeb1 expressions in A549 cells transfected with Opto-PI3K constructs or treated with TGF-β (10 ng/ml) alone or together with 100 nM wortmannin for 24 h. (**F**,**G)** The reversibility of Opto-PI3K induced loss of E-cadherin expression in A549 cells. Opto-PI3K expressing cells were exposed to blue-light LED array (0.2 mW/cm^2^) for different time points (Control, dark condition; LED ON 12 h, illuminated for 12 h; LED ON 24 h, illuminated for 24 h; LED ON 12 h/OFF 12 h, illuminated for 12 h then placed to dark for 12 h). After light treatment, cells were fixed and immunolabeled for E-cadherin expression. The transfected cells were identified with GFP signals, and untransfected cells were used as control. A549 cells were imaged with fluorescence microscopy (**F**) and the E-cadherin expression was quantitatively analyzed and plotted (**G**). **P < 0.01; Scale bar, 10 μm. Displayed blots are representative results of at least three independent experiments.

We next sough to test whether specific activation of PI3K in A549 cells induces EMT process, and to study how reversible controlled PI3K regulates EMT and cell function *in vitro*. The A549 cells were transfected with or without Opto-PI3K constructs, and stimulated with or without blue-light LED array (0.2 mW/cm^2^). As shown on Fig. 3A, we found that after 24 h of blue-light illumination, the Opto-PI3K transfected A549 cells displayed spindle-shaped morphology, whereas the control cells and transfected cells cultured in dark did not (Fig. 3A). These results indicated that sustained PI3K activation may induce EMT process in A549 cells, as the morphological changes on A549 cells with light stimulation were similar to TGF-β treated cells (Fig. 1A). To better investigate the effects of Opto-PI3K on induction of EMT in A549 cells, we carried out biochemical experiments and immunoblotted for two EMT characteristic markers, E-cadherin (E-cad) and vimentin expressions. After 6 h of transfection, the cells were illuminated with blue-light LED array (0.2 mW/cm^2^) as the time and conditions indicated (Fig. 3B,C). As shown on Fig. 3B, we found that sustained activation of PI3K by light potently decreased the expression of E-cad and concomitant up-regulation of vimentin expression on cultured A549 cells (Fig. 3B). As a control, the E-cad and vimentin expressions were unaffected in un-transfected control A549 cells with long-time light illumination at the same dose (Fig. S3B). To study whether the kinetics of PI3K activation affects EMT induction, we illuminated Opto-PI3K transfected cells with different doses of blue-light and probed for EMT markers expression. We adjusted the blue-light doses either by varying the time of illumination (Fig. 3B), or by changing the durations of ON/OFF frequencies in a total of 24 h illumination (Fig. 3C). Astonishingly, we found that Opto-PI3K induced regulation of E-cad and vimentin expressions were shown to be correlative with the illuminating blue-light doses, as both increasing the light exposure time and the frequency of light illumination (different ON/OFF cycles) accelerated the EMT process (Fig. 3B,C). The profound effect of Opto-PI3K in down-regulation of E-cad expression in A549 cells suggested that E-cad is an important downstream target of PI3K signaling (Fig. 3B,C). Previous studies showed that E-cad contributes to the activation of PI3K/Akt in ovarian cancer cells, and knockdown of E-cad expression was shown to inhibit PI3K/Akt signaling pathway in these cells^22,23^. On the contrary, in agreement with our findings, other groups found that E-cad was regulated by PI3K/Akt pathway, and constitutive activation of PI3K/Akt signaling was shown to down-regulate E-cad expression in cancer cells^24,25^. Our study provides the direct evidence that PI3K signaling regulates E-cad expression, as we revealed a light dose-dependent reduction of E-cad with Opto-PI3K activation (Fig. 3B,C).

Our data shown before demonstrated that it took several days to detect significant effects of TGF-β in regulation of E-cad and vimentin expression in A549 cells (Fig. 1B). To compare the effects of TGF-β and Opto-PI3K on this regulated process, we treated A549 cells with 10 ng/ml of TGF-β or stimulated cells with blue-light illumination (0.2 mW/cm^2^) for 24 h. Our results showed that Opto-PI3K had more potent effect on down-regulation of E-cad expression than 10 ng/ml of TGF-β treatment (Fig. 3D). As expected, both Opto-PI3K and TGF-β induced E-cad reduction were abolished by wortmannin (100 nM) treatment, suggesting an active PI3K signaling is necessary for this regulated process (Fig. 3D). Furthermore, we found that Opto-PI3K increased the expressions of Snail1 and Zeb1, the two EMT markers that work as repressors for E-cad expression^26^, suggesting their direct involvements in Opto-PI3K regulated E-cad down-regulation (Fig. 3E). Indeed, previous studies have showed that the expressions of Snail, Zeb and E-cad are inversely correlated. Overexpression of Snail1 and Zeb1 transcription factors *in vitro* induces loss of E-cad expression^27,28^. Furthermore, the Snail1 and Zeb1 expressions have shown to be regulated by NF-κB and GSK-3β signaling, whose activation can be modulated by PI3K/Akt signaling pathway and other TGF-β induced signaling cascades^29,30^. Thus, the involvement of NF-κB and GSK-3β signaling in Opto-PI3K induced E-cad reduction deserves further studies.

In addition, we took the advantage of optogenetics to reversibly activate PI3K and studied how that affected EMT in A549 cells. Opto-PI3K transfected cells were stimulated with blue-light LED array (0.2 mW/cm^2^) for 24 h, or alternatively the cells were illuminated with the same dose of light for 12 h and then recovered for another 12 h in dark condition. The A549 cells were fixed and E-cad expression in single cells was visualized by immunofluorescence staining. Our results demonstrated that Opto-PI3K induced EMT was reversible as we quantified E-cad expression after 24 h of treatment (Fig. 3F,G). The loss of E-cad expression induced by Opto-PI3K was recovered after we placed the A549 cells back into dark environment (Fig. 3G). The reversibility of EMT in cancer cells has been documented elsewhere^31,32^, but the mechanisms of its regulation have not been clearly studied. Previous studies showed that in the presence of prolonged TGF-β treatment, the cancer cells undergo three steady states as they distinguished with E-cad and vimentin expression features, which are E-cad^high^/vimentin^low^, E-cad^medium^/vimentin^medium^, and E-cad^low^/vimentin^high^, corresponding to the epithelial state, partial EMT state and full EMT state, respectively^31^. This research demonstrated that after removal of TGF-β for several days, the cancer cells in partial EMT state were able to reverse back to epithelial state^31^. Compared with this previous study, we think that the Opto-PI3K induced another uncharacterized EMT state, which can be defined as E-cad^low^/vimentin^medium^, and apparently from our results the A549 cells in this partial EMT state were highly reversible (Fig. 3B,G).

**Figure 4:**
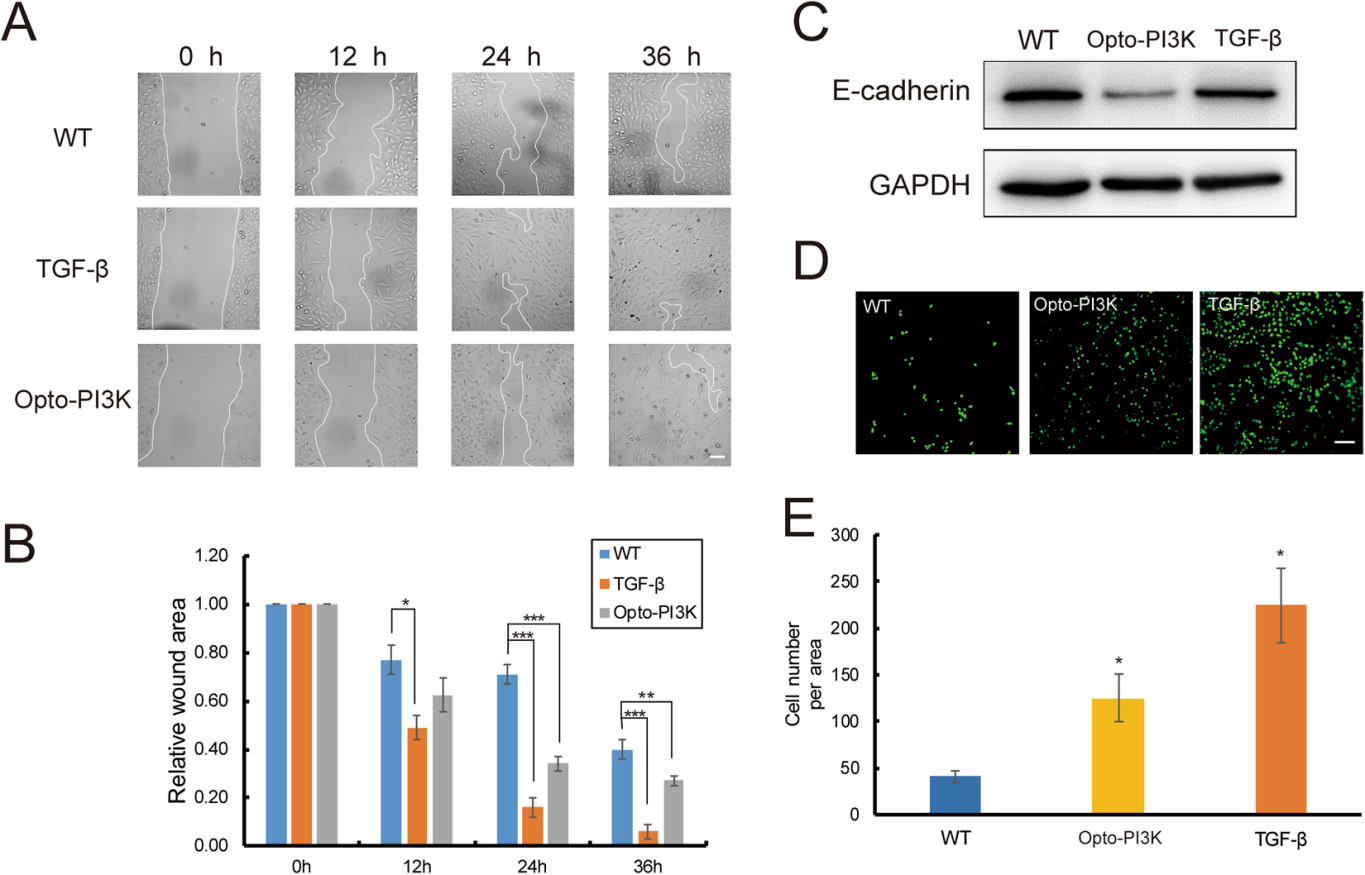
Opto-PI3K promotes cell migration in cultured cancer cells. (**A**) DIC images show the migration of A549 cells towards the wound area after scratch. A549 cells were transfected with Opto-PI3K constructs. The cells were treated with 10 ng/ml TGF-β or illuminated with blue-light LED array (0.2 mW/cm^2^) as the time indicated. The white line outlines the edge of cells. Scale bar, 100 μm. (**B**) Quantification of relative percentage of the normalized wound healing area at different time points after scratch. *P < 0.05; **P < 0.01; ***P < 0.001. (**C**) Immunoblot analysis of E-cadherin expression on HeLa cells stimulated with 10 ng/ml TGF-β or with Opto-PI3K activation for 24 h. Displayed blots are representative results of at least three independent experiments. (**D**,**E**) Transwell migration assay of HeLa cells stimulated with 10 ng/ml TGF-β or with Opto-PI3K activation. After 24 h of stimulation, the cells migrated through the pores were fixed and stained for nuclei. (**D**) Scale bar, 100 μm. Quantification of transwell migration was shown on (**E**). (*n* = 3 independent experiments, analyzed ~20 fields for each conditions; data are mean ± SEM). *P < 0.05 vs WT.

To further examine the function of this Opto-PI3K induced partial EMT in A549 cells, we performed *in vitro* migration experiments. The scratch-wound experiment demonstrated that both TGF-β (10 ng/ml) and Opto-PI3K significantly promoted cell migration towards the wound margin compared with untreated control cells (Fig. 4A). Interestingly, TGF-β displayed more potent effect on promotion of A549 migration than Opto-PI3K treatment, suggesting other signaling pathways stimulated by TGF-β are involved in this process (Fig. 4A,B). In addition, we performed transwell migration assay as another independent approach to evaluate the effects of Opto-PI3K on promotion of cancer cell metastasis on HeLa cells. Similar as what we detected on A549 cancer cells (Fig. 3B), we found that Opto-PI3K caused significant reduction of E-cad expression after 24 h of blue-light illumination (0.2 mW/cm^2^), whereas TGF-β (10 ng/ml) had barely effect (Fig. 4C). Transwell migration experiments showed that Opto-PI3K significantly increased HeLa cell migration through the pores, but its effect was less potent than TGF-β (10 ng/ml) stimulation (Fig. 4D,E). Collectively, these results indicate that Opto-PI3K partially mimics the effects of TGF-β stimulation on induction of EMT and promotion of cell migration on cultured cancer cells. These functional effects of Opto-PI3K induced cell migration and metastasis are likely to work through the E-cadherin-mediated pathway^33^, as we detected a strong reduction of E-cad expression after Opto-PI3K activation (Fig. 3B–D).

In summary, our study demonstrated the application of optogenetic PI3K module on cultured cancer cells, which enables specific and quantitative control of the intracellular PI3K activity. By precisely modulate the kinetics of PI3K activation, we found that E-cadherin is an important downstream target of PI3K signaling, and Opto-PI3K down-regulates E-cadherin expression in a light dose-dependent manner. Furthermore, we identified an uncharacterized partial EMT state regulated by sustained PI3K signaling, and A549 cells in this partial EMT state are reversible as demonstrated by optogenetic experiments. We further demonstrated that Opto-PI3K partially mimics the effects of TGF-β on promotion of cell migration, and we suspect that other TGF-β stimulated signaling pathways and their regulated negative feedback mechanisms on PI3K may contribute to the fully promotion of the irreversible EMT process on cultured cancer cells^34^. In addition, our established method of quantitative optogenetic control of intracellular PI3K signaling may have vast applications in cell biology research, and to explore other unidentified new roles of PI3K in cancer biology.

## Electronic supplementary material

Supplementary information
