# Supplementary material for: Optogenetic control of epithelial-mesenchymal transition in cancer cells
Source: Sci Rep. 2018 Sep 20;8:14098. doi: 10.1038/s41598-018-32539-3 (PMC6147862; doi:10.1038/s41598-018-32539-3)

Optogenetic control of epithelial-mesenchymal transition in cancer cells

Xiaoxu Zhou[1,¶], Jian Wang[2,¶], Junye Chen[1,4,¶], Yuankai Qi[1], Di Nan[1], Luhong Jin[1], Xiaohan Qian[1], Xinyi Wang[1], Qingyong Chen[2,*], Xu Liu[3], Yingke Xu[1,5,*]

[1]Department of Biomedical Engineering, Key Laboratory of Biomedical Engineering of Ministry of Education, Zhejiang Provincial Key Laboratory of Cardio-Cerebral Vascular Detection Technology and Medicinal Effectiveness Appraisal, Zhejiang University, Hangzhou 310027, China

[2]Department of Respiratory Oncology, The 117th Hospital of PLA, Hangzhou 310013, China

[3]Department of Optical Engineering, State Key Laboratory of Modern Optical Instrumentation, Zhejiang University, Hangzhou 310027, China

[4]Department of Hepatobiliary and Pancreatic Surgery, the Second Affiliated Hospital, School of Medicine, Zhejiang University, Hangzhou 310009, China

[5]Department of Endocrinology, The Affiliated Sir Run Run Shaw Hospital, Zhejiang University School of Medicine, Hangzhou 310016, China

Supplementary figures:

Figure S1. Blue light induced CIBN-CRY2 association in A549 cells. **A,** schematic drawing depicting constructs used to activate membrane targeting of mCherry-CRY2 to where CAAX-CIBN localizes. **B,** galleries of TIRFM images illustrate the dynamics of mCherry-CRY2 relocates to the plasma membrane after different doses of 488 nm laser activation. Scale bar, 10 μm. **C,** quantitative analysis of the dynamics of CIBN-CRY2 association upon different doses of 488 nm light activation ($t_{1/2}$=3.53±0.26s for 100mW; $t_{1/2}$=6.06±1.8s for 20mW; $t_{1/2}$=10.97±0.95s for 6mW; *n*=5 cells; Data are mean ± SEM).

Figure S2. Blue light induced PI(3,4,5)P_3 production in A549 cells. **A**, schematic of PI3K recruitment to the plasma membrane (CAAX-CIBN) using a fluorescent CRY2 fusion protein (mCherry–CRY2–iSH2) that constitutively binds to the endogenous PI3K catalytic subunit (p110α). Recruited PI3K converts PI(4,5)P_2 to PI(3,4,5)P_3 on the plasma membrane, which can be visualized by the PH-Akt–mRFP biosensor. **B**, galleries of TIRFM images visualize light induced PI(3,4,5)P_3 production on the cell surface. Scale bar, 10 μm. **C**, quantitative analysis of the dynamics of PI(3,4,5)P_3 production upon different doses of 488 nm light activation ($t_{1/2}$=21.31±4.94s for 100mW; $t_{1/2}$=33.7±5.56s for 20mW; $t_{1/2}$=41.31±9.34s for 6mW; *n*=5 cells; Data are mean ± SEM).

Figure S3. Prolonged illumination of A549 cells with blue-light LED array (0.2 mW/cm^2) had no apparent effects on cell function. **A**, immunoblot analysis of Akt phosphorylation on both Ser473 and Thr308 residues in A549 cells illuminated with or without blue-light LED array for 30 min. **B,** immunoblot analysis of E-cadherin and vimentin expression in A549 cells illuminated with or without blue-light LED array for 24 h. **C,** A549 cells were exposed to blue-light LED array illumination for 24 h. DIC images showed no apparent changes in cell morphology after blue-light exposure. Scale bar, 100 μm.

Figure S4. Repeated immunoblots data were shown for some quantitative plots in the main figures as indicated.

Figure S5. Raw immunoblots data were shown for some plots used in the main figures as indicated.

Fig.S1. Zhou, et al.

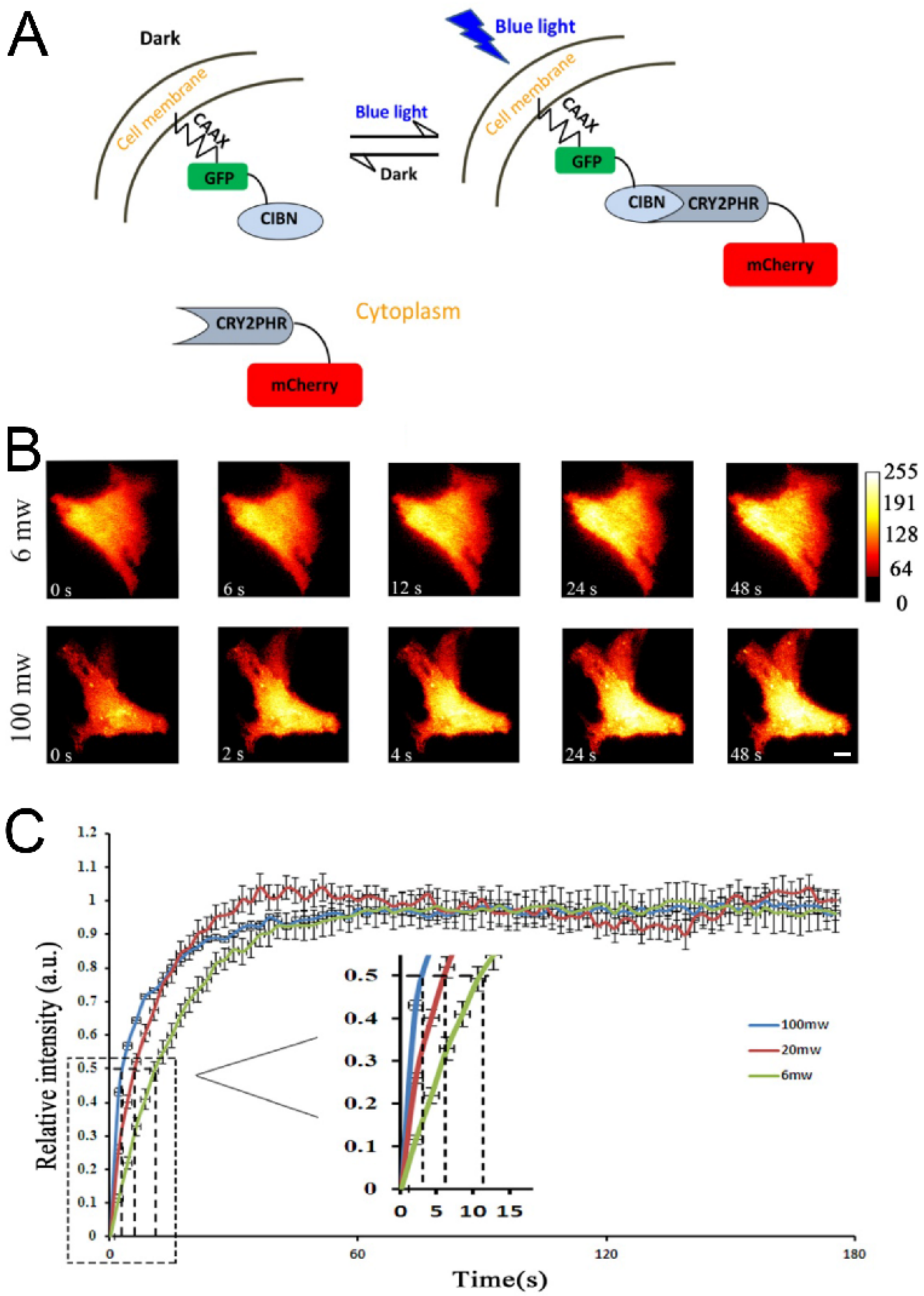

Fig.S2. Zhou, et al.

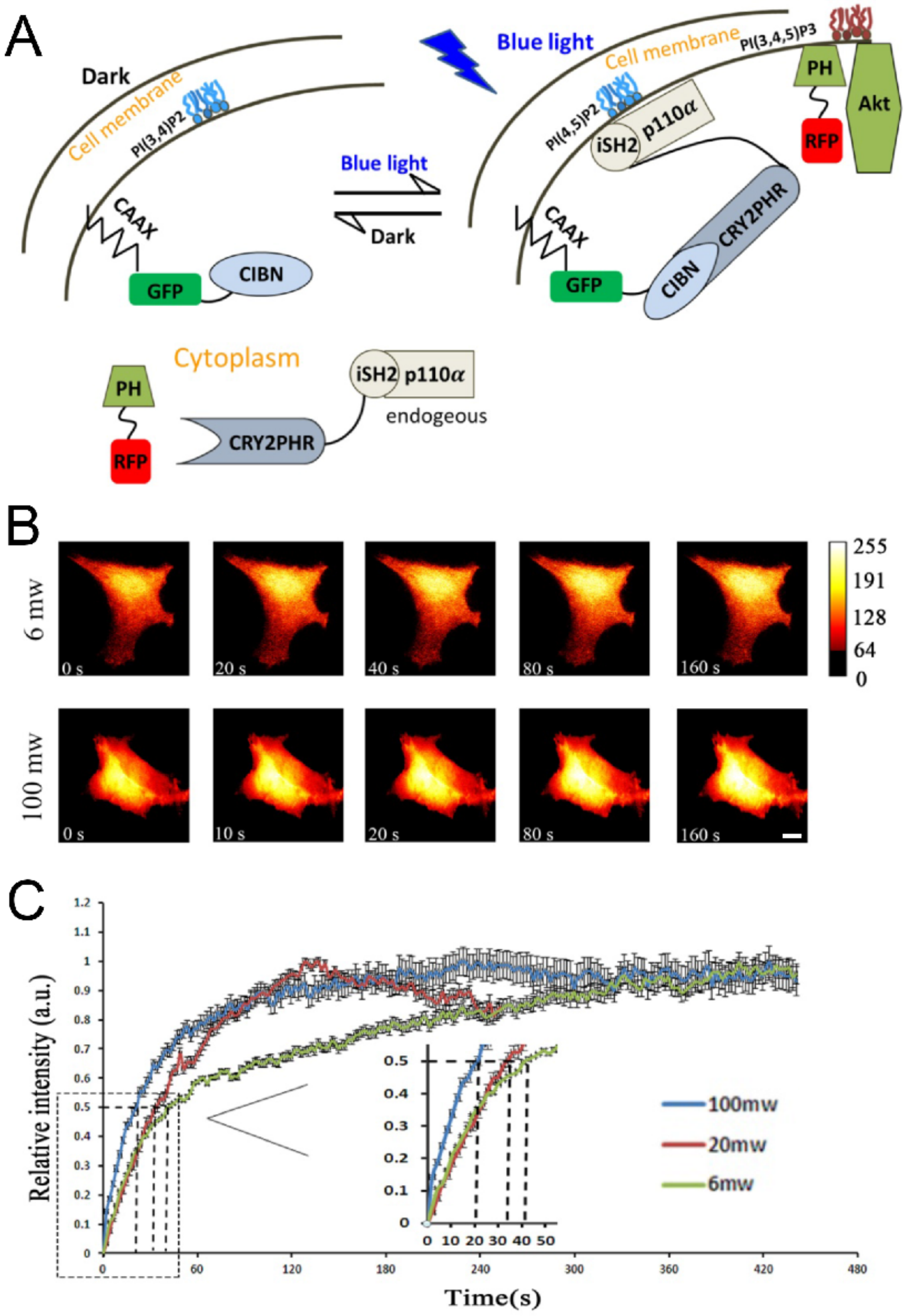

Fig.S3. Zhou, et al.

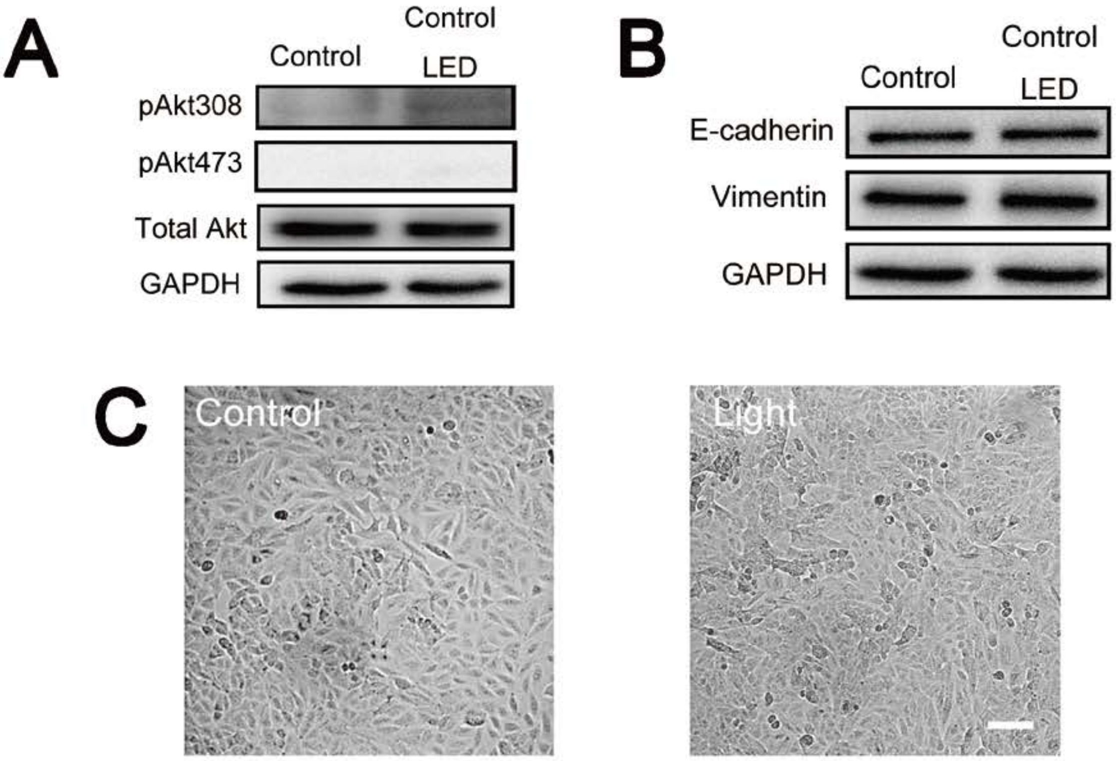

Fig.S4. Zhou, et al.

A. Repeated results of Fig. 1C&2E

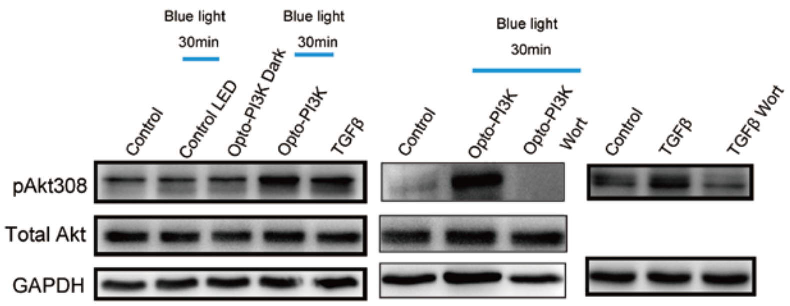

B. Repeated results of Fig. 2C

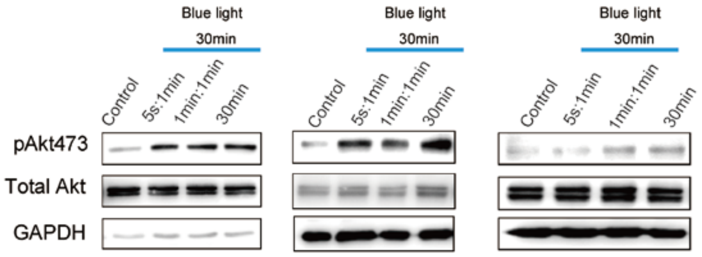

C. Repeated results of Fig. 2E

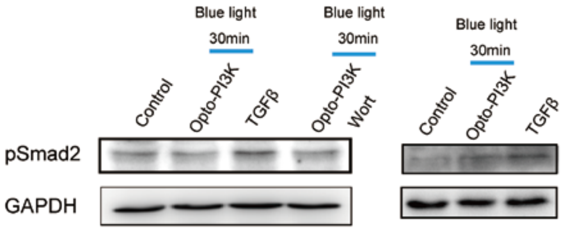

D. Repeated results of Fig. 3B&C

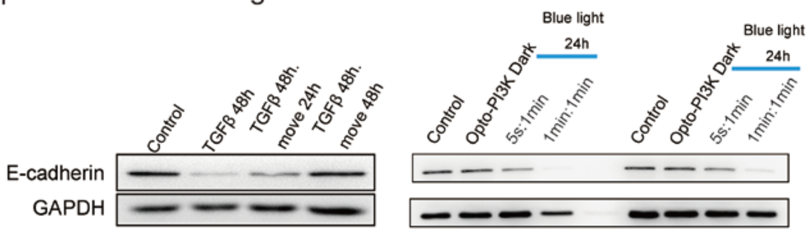

E. Repeated results of Fig. 3D

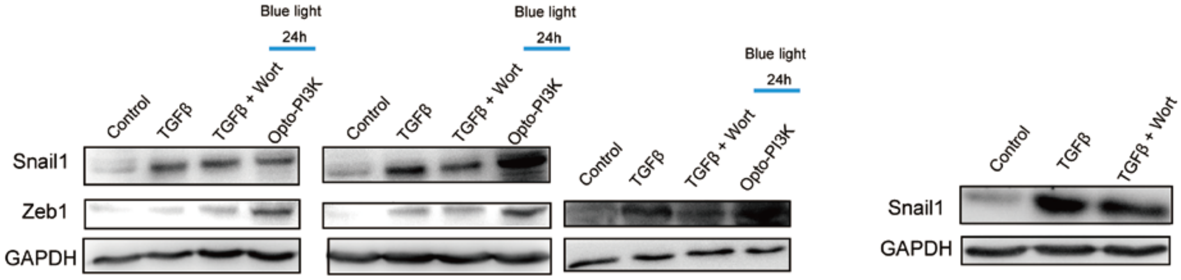

Fig.S5. Zhou, et al.

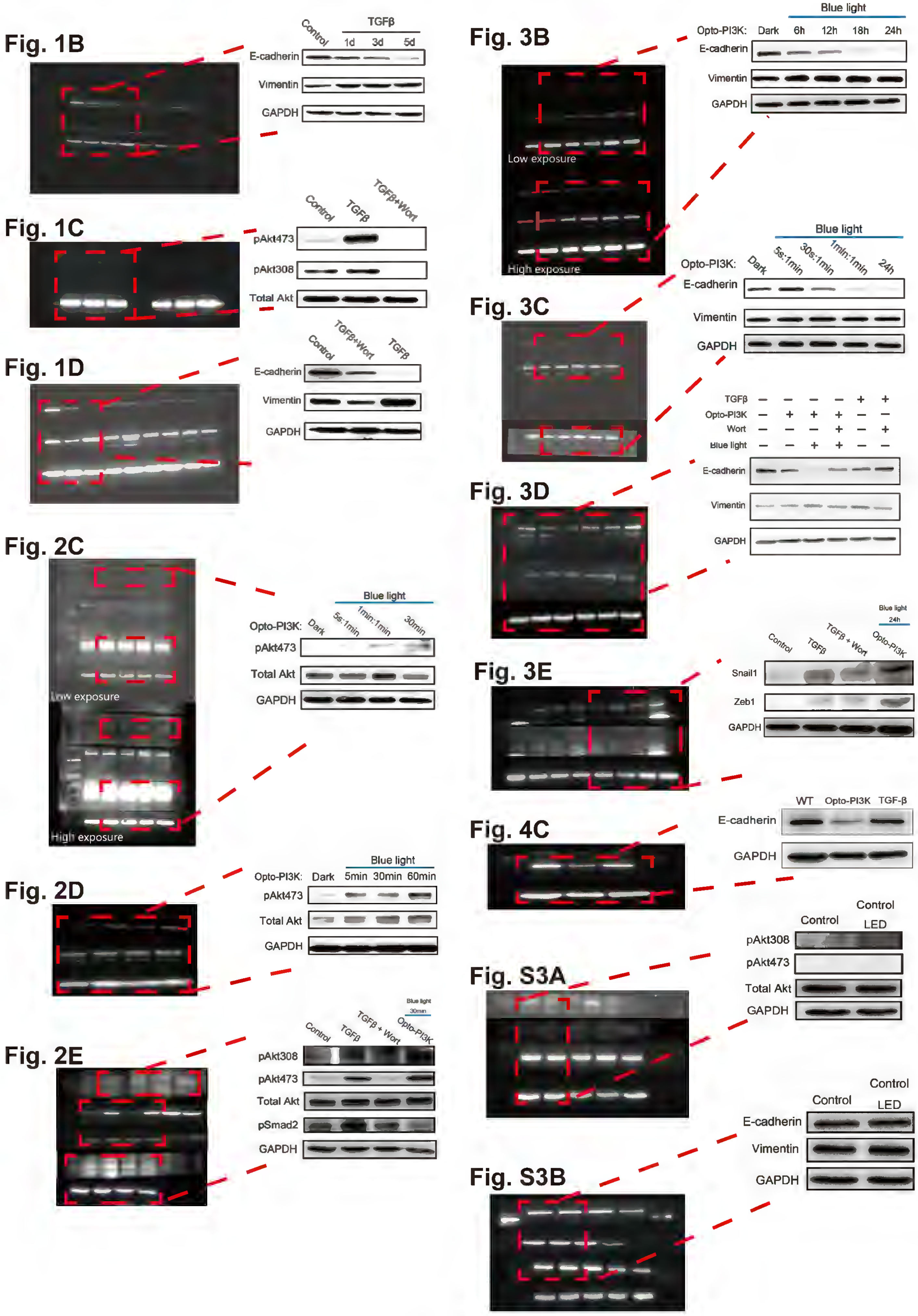

Supplement: Supplementary file 1 — Supplementary information [file 41598_2018_32539_MOESM1_ESM.pdf]
